# Exploring the molecular aspects associated with testicular germ cell tumors: a review

**DOI:** 10.18632/oncotarget.22373

**Published:** 2017-11-03

**Authors:** Gaetano Facchini, Sabrina Rossetti, Carla Cavaliere, Carmine D’Aniello, Rossella Di Franco, Gelsomina Iovane, Giovanni Grimaldi, Raffaele Piscitelli, Paolo Muto, Gerardo Botti, Sisto Perdonà, Bianca Maria Veneziani, Massimiliano Berretta, Micaela Montanari

**Affiliations:** ^1^ Progetto ONCONET2.0, Linea Progettuale 14 per l’Implementazione della Prevenzione e Diagnosi Precoce del Tumore alla Prostata e Testicolo, Regione Campania, Italy; ^2^ S.S.D Oncologia Clinica Sperimentale Uro-Andrologica, Dipartimento Corp-S Assistenziale dei Percorsi Oncologici Uro-Genitale, Istituto Nazionale Tumori “Fondazione G. Pascale”, IRCCS, Naples, Italy; ^3^ Medical Oncology Unit, ASL NA 3 SUD, Ospedali Riuniti Area Nolana, Nola, Italy; ^4^ Division of Medical Oncology, A.O.R.N. dei COLLI “Ospedali Monaldi-Cotugno-CTO”, Naples, Italy; ^5^ Radiation Oncology, Istituto Nazionale per lo Studio e la Cura dei Tumori “Fondazione Giovanni Pascale”, IRCCS, Naples, Italy; ^6^ Division of Urology, Department of Uro-Gynaecological Oncology, Istituto Nazionale Tumori “Fondazione G. Pascale”, IRCCS, Naples, Italy; ^7^ Pathology Unit, Istituto Nazionale Tumori “Fondazione G. Pascale”, IRCCS, Naples, Italy; ^8^ Scientific Management, Istituto Nazionale Tumori “Fondazione G. Pascale”, IRCCS, Naples, Italy; ^9^ Department of Molecular Medicine and Medical Biotechnologies, University of Naples “Federico II”, Naples, Italy; ^10^ Department of Medical Oncology, CRO Aviano, National Cancer Institute, Aviano, Italy; ^11^ Department of Biology, College of Science and Technology, Temple University, Philadelphia, USA

**Keywords:** TGCTs, epigenetics, chemosensitivity, cisplatin-associated resistance

## Abstract

Testicular germ cell tumors (TGCTs) represent the most common solid tumors affecting young men. They constitute a distinct entity because of their embryonic origin and their unique biological behavior. Recent preclinical data regarding biological signaling machinery as well as genetic and epigenetic mechanisms associated with molecular patterns of tumors have contribute to explain the pathogenesis and the differentiation of TGCTs and to understand the mechanisms responsible for the development of resistance to treatment. In this review, we discuss the main genetic and epigenetic events associated with TGCTs development in order to better define their role in the pathogenesis of these tumors and in cisplatin-acquired resistance.

## INTRODUCTION

Testicular germ cell tumors (TGCTs) represent the most common solid tumors affecting the young male population, with a peak incidence between the third and fourth decades of life [1]. TGCTs consist of several histological subtypes, including seminomas and non-seminomas, which derive from both gonadal and extragonadal anatomic sites [1, 2]. Seminomas and non-seminomas represent the two TGCTs main subtypes and have distinctly different biological features and metastatic potential, with non-seminomas showing extra-embryonal and embryonal differentiation patterns, including, embryonal-like somatic differentiated (teratoma), primitive zygotic (EC) and extra-embryonally differentiated phenotypes such as choriocarcinoma and yolk sac tumor, and driving a greater predisposition for early spread and a poorer prognosis in advanced stage disease [3]. On the contrary, seminomas show a highly sensitivity to both chemotherapy and radiation, with a good prognosis, while non-seminomas are sensitive to platinum-based combination chemotherapy and are less responsive to radiation, with the exception of teratomas [3]. Both seminomas and non-seminomas tumors show an invasive phenotype; they originate from a common ancestor, the carcinoma *in situ* (CIS), where tumor cells generation and expansion are confined to within the seminiferous tubules [4, 5]. Interestingly, non-seminomatous TGCTs include embryonal carcinoma (EC), which has similarities to stem cells and is able to differentiate into several somatic lineages whereas the cells that constitute seminomas tumors resemble to primordial germ cells (PGCs) and/or the cells in the CIS, making TGCTs an intriguing model for investigate gametogenesis and germ cell development in both normal and cancer systems. In fact, it has been reported that the initiating event in the pathogenesis of TGCTs occurs in utero during the embryonal development with the development of the intratubular germ cell neoplasia undifferentiated (ITGCNU), or CIS, which represents the first early lesion [4, 6], succeeded by a dormancy interval that terminates after puberty when postpuberal TGCTs emerge, suggesting that a hormonal event (hormone burst) could be responsible for the definitive occurrence of testicular cancer. However, a recent hypothesis relating to the genesis of testicular tumors and indicating the neoplastic cells, which retain stem cell features, as the origins of testicular tumors, has been reported [7]. Germ cell tumors often occurs as mixed tumors, which account for about 60% of cases, and include a combination of seminomatous and non-seminomatous elements, with two or more histological subtypes that are variably and randomly distributed throughout the tumor [4, 5].

Interestingly, despite differences in their specific cell of origin, TGCTs share the advantageous feature of being highly curable, due to their extraordinary responsiveness to therapeutic treatments [8]. Consequently, TGCTs, by representing an unique pathology at a cross-road in developmental and neoplastic processes, not only constitute a system for studying the mechanism of transformation of totipotent cells and their capability to differentiate into distinct germ lines but also a challenge for their exceptional molecular (genetic and epigenetic) features, responsible for their extraordinary sensitivity to chemotherapeutic drugs. Unfortunately, despite the great rate of cure, some of TCGTs are resistant to chemotherapy. Then, a greater knowledge of TGCTs biology can allow to achieve favorable progresses for the new therapies search and also to take advantage of this information for the therapeutic approach to other tumors. However, additional studies are needed since the molecular basis of TGCTs etiology and many aspects in the development of these tumors are still unclear. In the past years, genetic susceptibility, along with biological signaling machinery, and genetic and environmental factors, have been investigated in order to explain the mechanisms responsible for TGCTs susceptibility, transformation and development of resistance to treatment. This review will focus on the molecular events associated with TGCTs development in order to better define their role in the pathogenesis of these tumors and in cisplatin-acquired resistance.

### Etiology and risk factors

TGCTs have become important since they represent a major cause of death in the individuals between the ages of 15 and 35 years, covering approximately 1% of all cancers in men [1, 9]. Their incidence has been increasing worldwide over the past decade and their development has been associated with several urogenital abnormalities such as cryptorchidism (CO), hypospadias, and low fertility [10–12]. The incidence of TGCTs varies between different countries and races, being greater in Scandinavia, Switzerland and Germany than in Africa, Asia and Latin America, where a very low incidence has been reported, and in Caucasians-Americans compared to African-Americans [9]. The reasons for these differences in the incidence of TGCTs among different ethnic groups are unknown; however, it can hypothesize that the increased incidence of this disease in western countries is probably due to an increased exposure to etiologic factors. Moreover, both epidemiological and clinical data strongly indicate that environmental and genetic factors play a pivotal role in TGCTs genesis and development by altering PGCs normal differentiation processes. In fact, early stages of development (embryonic, fetal and infant) are particularly exposed to environmental events [13]. It has been proposed that genital malformations can be induced by intrauterine exposure to endocrine disruptors (EDs) during fetal development, and in young males [10, 14]. Recently, a link between the development of isolated TGCTs and certain risk factors, such as EDs, cryptorchidism, and family history of cancer, has been established in order to identify the key factors in testes carcinogenesis. Despite controversial results from some epidemiological studies, it has been proposed that dosage, exposure time, developmental stage of each individual, maternal lifestyle, genetic factors and the genetic variability of susceptibility to the exposure to EDs may be responsible for the EDs-induced damage [10, 14, 15]. Cryptorchidism has been proposed as a risk factor for the development of TGCTs, since patients, which are affected by CO, have a greater risk to develop TGCTs than the common population [10].

Different associations between TGCTs and prenatal factors have been investigated over the past years; none of these prenatal risk factors investigated have been strongly associated with the development of TGCTs, neither with other risk factors such as maternal smoking, preeclampsia, maternal and paternal age, maternal body weight, maternal parity, and trisomy 21 [16], although endogenous or exogenous hormone exposure during pregnancy, low birth weight and decreased gestational age are demonstrated to be associated with TGCTs [17]. Also, additional factors such as low maternal parity, twin birth, low birth order, breech presentation and breast feeding have all been studied as putative TGCTs risk factors [18]. However, the results obtained were conflicting and the evidences, at present, are still equivocal. The TGCTs relative risk is greatly increased in men with prior cryptorchidism, impaired spermatogenesis, testicular microlithiasis, testicular atrophy, and hypospadias [16, 19]. However, more than to predispose to cancer, these conditions are rather associated to it since they share the same etiologic factor. This is probably due Testicular Dysgenesis Syndrome (TDS), where altered Sertoli and Leydig cell function during testes development modifies the development of germ cells and leads to hypospadias, cryptorchidism, impaired spermatogenesis, decreased testosterone production, microlithiasis and testicular cancer [20]. Increased use of endocrine disruptors has been suggested to be one of the environmental factors responsible for the increasing incidence of testicular germ cancer cells in testicular dysgenesis syndrome [21, 22]. The EDs mechanism of action consists by the interruption in the endogenous hormones synthesis, release, transport, metabolism, binding, action or elimination during embryonic development before, during or after organogenesis [23]. The consequent variations in the hormone level can cause morphological and functional alterations in organisms. The risk of developing hormone-dependent cancers, such as the TGCTs, can be increased by small variations in estrogen levels during fetal development. Animal models have demonstrated that prenatal exposure to Bisphenol A (BPA), currently used in many products, such as baby and water bottles, and food containers, is associated with increased development of pre-cancerous lesions [24]. In addition, these animal models exposed to BPA during breastfeed have shown an increased risk of developing TGCTs, compared to unexposed animals [24]. Also, several studies have demonstrated a risk association between exposure to EDs in agricultural areas and genital abnormalities in males [25].

### Genetic abnormalities and polymorphysms associated with the pathogenesis of TGCTs

Different genes are involved in the pathogenesis of TGCTs; however, the role of genetic factors and their association with this pathology is still unclear. Interestingly, in recent years the findings regarding the association of different genes, (e.g. c-KIT/KITLG, POU5F1) with the development of this neoplasia, and the identification of aberrant epigenetic patterns in promoter regions of several genes, along with the expression of specific regulatory cluster (miRnas), have shed a better light in the comprehension of the development of this disease. Cytogenetic and molecular abnormalities are associated with TGCTs, and include: aneuploidy; the gain and/or loss of some specific chromosomal regions such as the presence of iso-chromosome 12p and the amplification of 12p sequences, which exist in all germ cell tumors and take place early during the malignant transformation; the gain of chromosomal material in 1, 2p, 7, 8, 12, 14q, 15q, 17q, 21q, and X or the deletion of chromosomal material from 4, 5, 11q, 13q, and 18q2 [5, 26]. However, the precise molecular mechanism(s) responsible for the carcinogenesis of germ cells and their progression is still poorly understood. Interestingly, it has been reported that 25% of TCGTs susceptibility are due by genetic effects [27]. However, genome-wide linkage analysis performed on familial TGCTs demonstrated no significant genetic linkage events, suggesting that several common, low-penetrance loci were responsible for TGCTs susceptibility [28]. In sporadic cases, the 1.6 Mb deletion in the AZF region of the Y chromosome represents the most common genetic alteration in patients with infertility and doubles the risk for developing TGCTs [29]. The strongest association for TGCTs susceptibility has resulted for single nucleotide polymorphisms (SNPs) at the 12q22 within the kit-ligand gene [30], which is correlated with a 2.5-fold increased risk of disease. This gene has been involved in several aspects of primordial germ cell (PGC) development and it seems to act on PGC survival and migration [31]. Strongly expressed in gonocytes in fetal and pediatric stages, in humans, the c-KIT gene encodes a tyrosine kinase activity receptor, and it is involved in spermatogenesis, hematopoiesis and melanogenesis [32, 33]. Its ligand KITLG (or stem cell factor, SCF), is located on chromosome region 12q21.3.2, which is necessary to carry out c-KIT dimerization and auto-phosphorylation, activating the c-KIT-KITLG signaling pathway and its downstream targets (e.i. k-ras) for proliferation and survival [34]. Variations in KITLG sequence (rs3782179, rs4474514 and rs995030) responsible for the predisposition to develop TGCTs have been recently documented, and also have been correlated with the role for KITLG in pigmentation, and with the greater incidence of TGCTs in Caucasian than in African-Americans males [17]. Moreover, the risk SNPs identified so far represent the most frequent alleles in Caucasian population, in general, and less frequent in the Black and Asian populations, possibly explaining TGCTs specific ethnic distributions. The KIT pathway has been suggested to be constitutively activated in human TGCTs as a result of gain of function mutations in the KIT oncogene and/or overexpression of KIT [34, 35]. In mouse models, KITLG germline heterozygous deletions induce an increase in TGCTs incidence [35].

Additional two genes identified along with c-kit as selected risk variants are SPRY4 and BAK1 genes. Interestingly, as c-kit/KITLG, these genes take part in germ cell survival and in early gonadal development [36, 37]. Located on chromosome 5, SPRY4 is an inhibitor of protein kinase pathway linked to TGCTs, whereas BAK1, which is located on chromosome 6, has been associated with this neoplasia since is a pro-apoptotic factor, whose expression is inhibited by KITLG-KIT pathway [38, 39]. Studies of single nucleotide polymorphisms in the gene regions of BAK1, DMRT1, TERTCLPTM1L, and KITLG demonstrated that these risk variants predispose to both bilateral and familial TGCTs [40]. Moreover, two independent signals within the TERT–CLPTM1L locus in 5p15, a locus on chromosome 12 containing ATF7IP, a regulator TERT expression 9p24 locus and containing the sex gene DMRT1 determiner were identified [41]. Located on chromosome 5, TERT-CLPTM1 is a transcription factor that controls ATF7IP and encodes for the telomerase catalytic subunit. In animal models, its loss is associated with progressive loss of male germ cells suggesting that TERT upregulation may be responsible for TGCTs development [42]. Interestingly, these identified risk SNPs are independent of the presence of cryptorchidism, spermatogenic function as well as familial predisposition [43, 44]. In addition, in PTEN a SNPs only (rs11202586) has shown association with the risk of developing TGCTs, regardless of histological subtype and hereditary factors [45]. Moreover, additional (meta-) analyses have been identified several alleles risks related to UCK2 [46], HPGDS, RFWD3, MAD1L1, and RAD51C, TEX14, and PPM1E [47], and PRDM14, DAZL, and PITX1 [48]. Interestingly, a significant association between the risk of developing TGCTs and UCK2 gene on chromosome 1q23 has been identified [46]. Recently, a polymorphic p53 response element in KITLG has been found to be linked to the TGCTs development [49]. This is of considerable interest since point mutations are rare events in testicular germ cell tumors and P53 results no mutated in these tumors.

Another important factor of TGCTs development is sex steroids, which play an important role in the risk and progression of malignancy. Polymorphisms in 17-β hydroxydehydrogenase-4, the enzyme responsible for the androgen to estrogen conversion, have been described and they have been associated with TGCTs [50, 51]. In addition, polymorphisms in cytochrome P450 Cyp-1A1 gene, encoding a hormone-metabolizing protein, have been identified and correlated with susceptibility to TGCTs development [52, 53]. Since the classical genomic androgen action is mediated by the androgen receptor (AR), one of the most studied genes in regards to polymorphisms is the AR. The AR gene is located in the Xq11q12 region and it presents two polymorphic regions, located in the trans-activation domain, with CAG and GCN codon (that encode for glutamine and glycine, respectively) [54]. Changes in the length of these polymorphic trinucleotide repeats lead to AR altered transactivation, and it has been reported as strongly associated with the increased risk to develop seminoma, suggesting that AR increased transactivation may occur in the development of seminoma and/or in the progression of carcinoma *in situ* to seminoma [55]. It is postulated that the presence of these polymorphic sequences may be involved in increasing the risk of TGCTs, since these variants alter receptor function that leads to insensitivity of androgens, causing high concentrations of testosterone and estrogen in circulation. Moreover, specific SNPs (P390S, A279T, rs12014709) have been identified and associated with TGCTs development [56]. DMRT1 is a transcription factor, belonging to the DNA binding gene family, which has strong implications in testicular development in vertebrates. Expressed as a pluripotency gene in TGCTs, like other genes as POU5F1 and NANOG, it takes part in regulating gametogenesis, differentiation, sexual determination and in tumor pluripotency [57, 58]. Located on chromosome region 9q24.3, DMRT1 is also involved in tumor development, with its genetic variants (rs755383 and rs7040024) having a strong relationship with susceptibility to develop TGCTs [40, 59, 60].

### Epigenetic events in the development of testicular germ cell tumors

Although several studies have tried to elucidate the exact role of the various factors responsible for TGCTs development, the molecular mechanisms underlying this disease still need to be elucidated. It is therefore likely that, along with genetic factors, epigenetic mechanisms (i.e. aberrant DNA methylation) can represent an alternative pathway that could explain the etiology of this disease. In fact, epigenetic regulatory processes occur in both the mechanisms of initiating and protecting pluripotency of embryonic stem cells as well as in maintaining the identity of differentiated cell types [61]. Deregulation of these processes may change chromosomal stability, stem cells properties, self-renewal and the potential to differentiate, leading to initiation and/or progression of cancer, including testicular cancer. Thus, altered or dismantled epigenetic regulation might therefore be one of the underlying factors in the origin and biology of TGCTs. In cancers, alterations in gene methylation in relation to tumor suppressor genes have been shown to be common and represent an important step in tumorigenesis [62]. Aberrant hypermethylated CpG islands have been identified in about every tumors, including TGCTs [63]. Interestingly, TGCTs uncommonly show tumor-related genes aberrant methylation. DNA hypomethylation of oncogenes leads to DNA overexpression that, in turn, may result in carcinogenesis [63, 64]. Moreover, aberrant DNA methylation and overexpressed DNA methyltransferases (DNMTs) were observed in TGCTs or its subtypes. While seminomas are distinguished by a global hypomethylated genome, the more differentiated non-seminomas (teratoma, yolk sac tumor, and choriocarcinoma) display a hyper-methylated genome, similar to somatic tissues [65–67]. Interestingly, embryonal carcinoma cases showed an intermediate methylation pattern [68]. However, a small part of the seminomas, GCNIS and gonadoblastoma show a high DNA methylation level, similar to that observed in the non-seminomas [65–67, 69]. Staining for methylation at the 5 position of deoxycytidine residues (5mC) was markedly reduced and virtually undetectable in the majority of ITGCN and seminomas. On the contrary, a notable staining was observed in non seminoma tumors [67].

Aberrant methylation of the regulatory genes promoter region silences their expression, representing a critical pathway in the development of cancer [70]. Hypermethylation of CpG islands located in the tumor suppressor genes or tumor-related genes promoter regions is considered as a key mechanism for gene inactivation [71]. However, tumor suppressor genes or tumor-related genes aberrant de novo methylation is a rare event in TGCTs respect to testicular malignant lymphomas [72]. Moreover, genes, which in adult human cancers are frequently methylated at promoter CpG islands, are generally unmethylated in both embryonic stem cells and embryonal carcinomas cells [73]. Epigenetic studies of sporadic cases of TGCTs have show that DNA methylation is critical for germ cells development, and these enzymatic modifications depend on DNMTs. Specifically, DNMT3a and DNMT3L isoform, are responsible for de novo methylation during germ cell development in the prenatal stage, while DNMT1 and DNMT3b occur after birth in the male, and are all involved in the maintenance of methylation patterns in spermatogonial proliferation [74].

Not expressed in seminoma, DNMT1 results upregulated in embryonal carcinoma, whereas DNMT3a is up-regulated in TGCC compared to non-tumor testicular tissues [75, 76]. The expression pattern of DNMT3b has been deeply investigated; it could be considered as a predictive marker for an increased risk of relapse in patients with stage I seminomas, and it is correlated with other pathologic features of poor prognosis (i.e. tumor size, rete testis invasion or vascular and lymphatic invasion) [77]. Highly expressed in EC cells, DNMT3b may be partially responsible for the hypersensitivity of these cells to second generation demethylating agent guadecitabine [78].

Overexpressed in the non seminoma tumors, DNMT3L represents a novel factor, crucial for the growth of human EC since its silencing in these cells results in growth inhibition with consequent increase of LINE1 sequences methylation [79]. Thus, the difference in the degree of methylation between seminomatous and non-seminomatous TGCTs mostly derived from the degree of DNA repetitive elements methylation, particularly at Alu elements [80]. The degree of demethylation of the repetitive elements is more pronounced in seminomas compared to non-seminomas, and GCC were more demethylated compared to cancers originating from somatic tissues [80]. In addition, the degree of LINE1 and Alu demethylation in TGCTs was more pronounced than that of cancer cells of somatic tissue origin (i.e.testicular malignant lymphoma and renal cell carcinoma). Interestingly, these unique methylation patterns of DNA repetitive elements, existing in TGCTs, in seminomas most likely reflect the origin of TGCTs and their pluripotent nature rather than global DNA demethylation often observed in cancer. However, both in seminomas and non-seminomas tumors, the LINE-1 DNA hypomethylation may be also due to epigenetic inactivation of PIWI-interacting RNAs (piRNAs), a class of small non-coding RNAs, predominantly expressed in the germ cell lineage and transcribed from genome regions containing transcribed transposable and other repetitive elements, and different Argonaute protein family members (PIWIL1, PIWIL2, PIWIL4) [81, 82].

Early fetal germ cells and undifferentiated germ cell tumors have in common the expression of pluripotency markers such as the transcription factors Nanog and Oct3/4. Nanog, a protein reciprocally regulated by Oct4 and p53, is a homeobox-containing transcription factor and a core pluripotency network member itself, and also is a key regulator of self-renewal and maintenance of pluripotency in undifferentiated embryonic stem cells and in suppression of differentiation [83, 84]. Detectable in germ cells, seminoma, embryonal carcinoma and carcinoma *in situ*, Nanog expression is not detectable in the adult testis or in differentiated somatic cells and its promoter resulted hypomethylated in spermatogonia and hypermethylated in sperm [85]. OCT3/4-SOX2 mediated expression of Nanog can be silenced by methylation of promoter CpG-sites [85]. In humans, DNA methylation of distinct promoter elements (NRR) CpGs is able to epigenetically induce the silencing of Nanog, expression. Furthermore, in fetal germ cells, adult testis tissue and mature sperm, Nanog, expression correlates to NRR methylation whereas in spermatogonia NRR remains hypomethylated but NANOG is not expressed due to lack of the expression of its mediators (OCT3/4-SOX2) [85]. Thus, in sperm and in adult testes NRR-hypermethylation could represent a tool by which NANOG expression could be epigenetically repress then controlling the pluripotency program and preventing germ cell malignancies.

Aberrant DNA methylation can provide an alternate genetic mechanism for susceptibility to familial TGCTs. In primary lymphocytes elevated promoter methylation of PDE11A, SPRY4 and BAK1, and lower KITLG promoter methylation, are linked to familial TGCTs risk [86]. Here, these changes in promoter methylation may inactivate PDE11A, SPRY4 and BAK1 and potentially activated KITLG and then the KIT pathway [86]. Thus, promoter methylation of these genes can alter familial TGCTs risk in a way compatible with the influence exerted by these genes variants on TGCTs risk. In non seminoma tumors several promoter genes (i.e. BRCA1, RASSF1A, MGMT, HIC1, APC, FHIT) are hypermethylated [66]. Interestingly, sensitive tumors showed hypermethylation of MGMT (which is involved in DNA adduct removal) and RARB (involved in RA signalling), whereas resistant tumors had hypermethylated RASSF1A and HIC1 promoters [66].

Epigenetic modifications are also carried out in spermatogenesis by several members of the histone methyltranferases family (HMTs), which may mediate the dimethylation or trimethylation in histone 3 (H3) of lysine 9. It has been suggested that dimethylation of arginine 3, histones H2A and H4 may be a mechanism by which seminomas and CIS/IGCNU maintain their undifferentiated state; while the loss of these histone modifications could be involved in somatic differentiation observed in no seminoma tumors [87]. p63 and p73, two p53 family members, might play a role in germ cell tumor cells. Interestingly, a p63 isoform (GTAp63), which is uniquely expressed in the testis of humans and great apes, is uniformly expressed in CIS cells, although a loss of expression, due to epigenetic regulation, has been observed in about 70–100% of all invasive tumour cells, leading to the hypothesis that in germ cells this protein may act as a tumour suppressor [88]. In non-seminoma gene activating histone methylation H3-K4 and gene silencing histone methylation H3K9 has been detected in all histological subtypes suggesting that these events could be associated with abnormal gene expression in this testicular tumor subtype [89]. Furthermore, in carcinoma *in situ* low levels of repressive histone modifications at H3K9me2 and H3K27me3 along with high levels of H3K9 acetylation and H3K4 methylation exist [90].

### Molecular mechanisms responsible for chemotherapy sensitivity and resistance in TGCTs

Due to their extremely high 5-years survival rate and to the efficacy of cisplatin treatments to cure more than 80% of metastatic testicular cancers [91], TGCTs are considered as curable neoplasms. However, the development of chemoresistance have occurred also in these tumors prompting several authors to focus their studies not only on the molecular events responsible for these resistance mechanisms but also on those resulting in TGCTs high chemotherapy sensitivity in order to develop more effective treatment for patients with metastatic cancers of somatic tissue origin. Moreover, the precise description, at the molecular level, of the mechanisms responsible for chemosensitivity may provide a deep comprehension of the chemoresistant TGCTs and, new specific tools for therapeutic interventions intended to revert them to chemotherapy responsiveness.

Chemosensitivity is an intrinsic state of the tumors, which is not related to a specific drug or drug combination utilized, but depends on its ability to sense damage, to activate the DNA damage response (DDR) and to respond by undergoing apoptosis instead of cell cycle arrest and DNA damage repair. In TGCTs, complex DNA rearrangements occur especially in their DNA machinery repair, where the decreased repair ability, due to their low DNA repair protein expression levels, render these tumor cells more sensitive to the drug [92, 93]. Furthermore, non seminomas primary tumors, who generally exhibit a higher resistance to chemotherapy, show a higher ERCC1 (excision Repair Cross Complementation group 1) protein expression compared with seminoma tissues [94]. Moreover, increased expression of this protein has been shown in both cisplatin resistant cell lines and primary TGCTs specimens compared to their respective cisplatin-sensitive counterparts [95]. Interestingly, although the down-regulation of the DNA repair elements may contribute to chemosensitivity, the way DNA damage is sensed by the cell and the p53 response to it both seem to account for chemotherapy responsiveness.

Frequently mutated in about half of all human cancers and functionally inactivated through non-genomic mechanisms in the remaining malignancies, the tumor suppressor p53 is not mutated in TGCTs, and is activated following exposure to chemotherapeutic agents, both events that have implications for the chemosensitivity of these tumors [96, 97]. Normal p53 activates two main distinct and mutually exclusive cellular programs, leading to apoptosis and to cell cycle arrest, respectively [98]. Then, in tumors as TGCTs that retain wild type p53, any p53 activity, which remains after non-genomic neutralization, could become associated with drug resistance when the cell cycle arrest program is activated or with treatment sensitivity when this remaining activity proceeds forward the apoptotic program. In fact, in these tumors their inability to repair affects, in turn, the program activated downstream of p53, given that increased damage burden promotes apoptosis over cell cycle arrest [99, 100]. Several studies have confirmed that apoptosis plays a pivotal role in the exceptional TGCTs sensitivity to cisplatin treatments, due to their unique sensitivity to p53 activation. In TGCTs wild-type p53, whose silencing can completely abolish the sensitivity to cisplatin, decides between cell cycle arrest and apoptosis, leading to resistance and chemosensitivity, respectively. Several factors can determine which p53 program will be activated within a cellular context. In addition, the nature of the activation signal, the presence of concomitant signals and p53 post-translation modifications (e.g. phosphorylation, ubiquitination, SUMOylation, acetylation), can decide whether a cell will undergo apoptosis or cell cycle arrest following p53 activation [97, 101]. Specific p53 modifications have been associated with the execution of apoptosis versus cell cycle arrest: the phosphorylation of p53 serine at position 46 by kinases DYRK2 and HIPK2 kinases, and the acetylation of two lysine residues K120 and K320 located in the DNA binding domain and in the tetramerization domain of p53, respectively, promote cell cycle arrest over apoptosis [102–105]. Interestingly, in TGCTs p53 is not only functional but also increased in amount, which denotes that either its production is increased or its degradation is decreased [106]. Also, in these tumors the enzymes serving apoptosis promoting p53 post-translation modifications are overexpressed or up-regulated and the enzymes that are involved in p53 alteration promoting cell cycle arrest are suppressed or down-regulated having consequences on the choice of the transcriptional p53 program which is triggered following activation. Increased transcription of genes such as PUMA, NOXA and p53AIP1 activates the apoptotic program whereas increased transcription of genes such as p21, GADD45 and TIGAR (TP53 Induced Glycolysis and Apoptosis Regulator) are responsible for the promotion of cell cycle arrest program [107, 108]. Interestingly, cisplatin-resistant TGCT cells retain wild type p53 but, in contrast to sensitive TGCT cells, activate p21 and HDM2 expression after drug treatment [109]. Interestingly, p53 can interact with Oct4 leading to an interesting interplay between these two factors responsible for the chemotherapy response of these tumors [8, 110].

**Figure 1:**
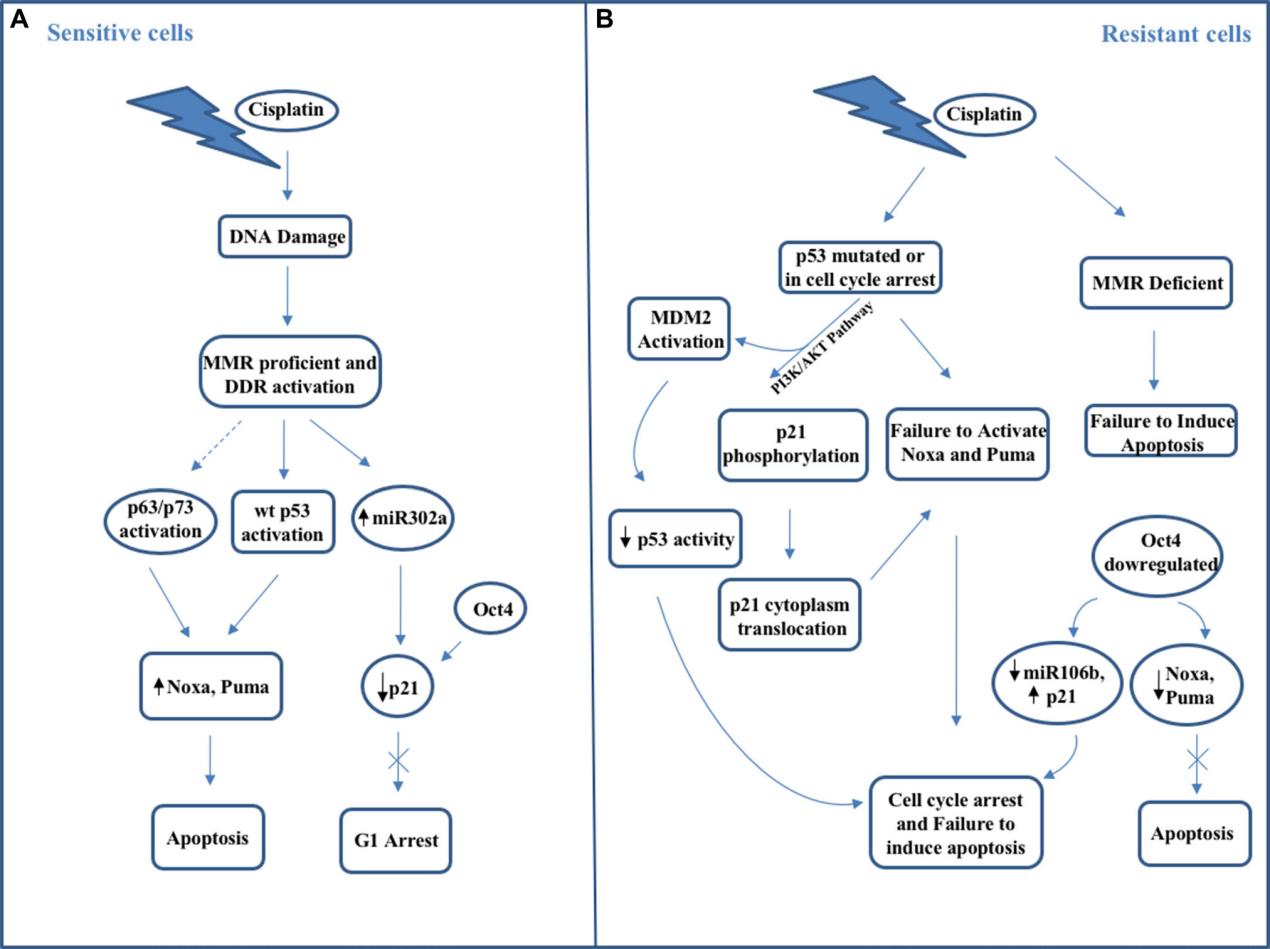
Schematic representation of main molecular mechanisms occurring in (**A**) cisplatin-sensitive testicular cancer cells and (**B**) cisplatin-resistant testicular cancer cells.

Oct4 (also called OTF3 or POU5F1), an embryonic transcription factor that binds to the octamer DNA sequence ATGC(A/T)AAT through its POU domain, is expressed in embryonic stem cells where it controls their survival and also their pluripotency by cooperating with different transcription factors such as Sox2 [111], and it is also uniformly expressed in the seminoma and embryonic carcinomas [112]. In germ cells Oct4 expression is induced by Leydig cell-associated signaling via the IGF-1 (Insulin-like Growth Factor 1) and PI3K/Akt pathways [113]. Phosphorylation of Oct4 by Akt promotes its interaction with Sox2 on genes target promoters and inhibits Oct4 ubiquitination leading to degradation of unphosphorylated Oct4 from the Akt promoter [114]. In TGCT cells, hypoxia promotes Oct4 SUMOylation at lysine K123 and Oct4 consequent down-regulation, an event responsible for cisplatin and bleomycin resistance [115]. On the contrary, peptidase SENP1 can de- SUMOylate Oct4 and improve the chemosensitivity of TGCT cells [115]. Interestingly, following p53 activation, re-expression of Oct4 in seminomas and the embryonal carcinomas may contribute to induction of apoptosis in these cancers [110, 111] and prevent p53-induced cell cycle arrest and differentiation. In TGCT cells the reciprocal regulatory mechanisms by which Oct4 respectively suppresses/induces p21 and Nanog, that in turn, are respectively activated/suppressed by p53 are then critical for determining chemotherapy responsiveness since their espression profile is correlated with therapy response or resistance [110, 116, 117]. Lack of Oct4 expression in embryonal carcinoma cells correlates with cisplatin resistance [118]. Also, these cisplatin resistant cells display a significant p21 expression, which is absent in seminomas [119]. In addition, primary embryonal carcinomas from patients that were cisplatin-sensitive display high Oct4 and no p21 expression, whereas patients with cisplatin-resistant mature teratomas resulted negative for Oct4 expression and strongly positive for p21 expression [120]. In other cancer types, increased expression of Oct4, along with other stem cell factors, is associated with cisplatin resistance [121]. Thus, probably the TCGCs specific cellular context rather than the presence of Oct4 per se is responsible for promoting chemosensitivity in these tumors. In fact, Oct4 is a stemness, prosurvival factor for normal germ cell progenitors, which, following chemotherapy, become a pro-apoptotic factor. Probably, in TGCTs the absence of p53 mutant deprives the respective cancer cells from several cancer promoting properties such as the ability to induce transcription from high affinity promoters (i.e. p21 promoter). The presence of p53 mutant has been proposed to enhance reprogramming of normal cells to pluripotent stem cells in the presence of Oct4 and Sox2 [122]. Thus, in TGCTs, the absence of p53 mutations may be consistent with, or even imposed by, Oct4 (normally expressed in germ cell progenitors) (re)expression in order to support carcinogenesis by establishing the pro-survival embryonic network that is nowa typical feature of these tumors. Then, it is not the p53 or Oct4 expression itself that leads to chemosensitivity of TGCTs, but rather the p53-Oct4 interplay. The balance between these two factors, one (Oct4) as a member of the core factors of stemness and an integral component of the recently proposed reprogramming of somatic cells to induced pluripotent stem cells, and the other (p53) responsible as blocker of pluripotency induction, negates the effects that both have on promotion of differentiation, cell cycle arrest, establishment of stemness and promotion of survival, and it is responsible for retaining the sensitivity to apoptotic stimuli. Conversely, both mutations in p53 and loss of Oct4 expression lead to cisplatin resistance [110, 123, 124] (Figure 1). In these opposing actions, several targets of p53 and Oct4 play an important role in cell cycle arrest. Among them, the CDK inhibitor p21, which is induced by p53 and down-regulated by Oct4, has also a role in the suppression of pluripotency by applying a brake to the rapid cycling of self-renewing progenitors [125]. In TGCT cells, following cisplatin treatment, down-regulation of p21, cell cycle arrest in G2/M phase and apoptosis occur as results of miR-302a up-regulated expression [126]. In addition, miR372 and miR373, induced by Oct4, Sox2 and Nanog and frequently overexpressed in TGCTs, by suppressing the expression of LATS2 (Large Tumor Suppressor homolog 2), a target gene for induction by wild type p53 and for reciprocal regulation by p53/Oct4, interfere with cell cycle arrest p53-p21 mediated and increase CDKs activity, leading to uncontrolled cell proliferation [127]. Thus, p53 and Oct4 have both individual effects that may result in chemoresistance, but their combined action leads to sensitivity for apoptotic stimuli. Therefore p53 is not the unique factor that determines cisplatin responsiveness. Probably, p53 family members like p63 or p73 could play a role in TGCTs which have lost functional p53, i.e. by regulating Puma and Noxa [88, 128], or other p53 regulator such as MDM2 may intervene [110, 129] (Figure 1). In TGCTs, the association of DNA mismatch repair (MMR) function with chemosensitivity may relate to low NER (nucleotide excision repair) activity. However, resistant tumors were frequently found to be associated with defects in MMR and to exhibit microsatellite instability (MSI) resulting from a low expression of the MMR proteins MLH1, MSH6 and MSH2 [130, 131]. If MMR function is lost, additional lesions such as BRAF activating mutations are required for the cells to remain viable, and these may lead to concomitant chemoresistance [130]. Thus, the responsiveness to therapeutic treatments inducing DNA damage is due to the lower threshold to undergo apoptosis following DNA damage, the decreased capability to repair cisplatin-induced DNA damages and the lack of active pumps able to transport cisplatin outside the cell [131, 132]. Also, the reduced capability to repair damaged DNA is due to the errors existing both in homologous recombination and in inter-strand crosslink repair [133]. In addition, in TGCTs elevated wild-type p53 protein intratumoral levels are responsible for upregulating pro-apoptotic factors such as Noxa, Puma and Fas [133]. Moreover, the expression of numerous genes responsible for controlling G1/S phase checkpoint and/or allowing apoptosis, such as FASLG, TNFSF10, and BAX, is up-regulated [110] in TGCTs, where high expression of BCL2- associated X protein, and low levels of the anti-apoptotic protein BCL2 exist [134]. However, about 20% of TGCTs patients becomes cisplatin-refractory or, after a first positive response, aquires resistance to cisplatin-based chemotherapy. Most of the hypotheses explaining the mechanisms of cisplatin resistance in TGCTs have focused on DNA mismatch repair pathway, which in chemoresistant TGCTs is presented as impaired causing failure to carry out apoptosis and then leading to cisplatin resistance [135]. Interestingly, mismatch repair factors have resulted hypermethylated in these cells [130]. In addition, an association between the global DNA methylation status and the response to chemotherapy has been proposed, suggesting that a degree of methylation may dictate the chemosensitivity or resistance in TGCTs. DNA hypermethylation has been also strongly correlated with microsatellite instability and mutated BRAF V600E, two genetic anomalies often present in resistant tumors and linked to poor outcome [130]. Nevertheless, a different study have shown that no BRAF V600E mutations have been found in resistant TGCTs although other somatic mutations (PIK3CA, AKT1, KRAS, HRAS, FGFR3) have been identified in resistant TGCTs [136]. Interestingly, for the first time, FGFR3 is resulted as the most frequently mutated gene; however, the mutations occurring in FGFR3 have not been correlated to TGCTs cisplatin sensitivity or resistance [136]. On the contrary, all observed AKT1 and PIK3CA mutations resulted to be mutually exclusive and only present within cisplatin-resistant tumors [136]. These findings are important since they suggest that PIK/AKT pathway activation is one of the main mechanisms of cisplatin-resistance and a potential future therapeutic target. Phosphorylated AKT (pAKT) was recently demonstrated to play a role in cisplatin resistance since in TGCTs induces the shuttling of p21 to the cytoplasm where the CDK inhibitor binds to cyclin-dependent kinase 2, thereby inhibiting the apoptotic response to cisplatin-induced DNA damage [110, 118]. Inhibition of pAKT either directly or by blocking PI3K signaling resulted in p21 nuclear re-localization and then in the reversal of cisplatin resistance with a remarkable increase in apoptosis [110, 118]. OCT4 and microRNA-106b alter the cytoplasmic p21 expression since their high expression level is correlated with p21 low expression, offering in turn a greater sensitivity to cisplatin-based therapy in testicular cancers [110, 118]. Despite the presence of high wild-type p53 levels, in TGCTs the resistance to platinum agents can probably be due to the persistence of p53-MDM2 complex in cisplatin-resistant TGCTs [137]. Consequently, in chemo-sensitive and chemo-resistant testicular cells, the direct targeting of cytoplasmic p21 or the Oct4/miR-106b/p21 pathways modulation or the inhibition of MDM2-p53 interaction leads to a p53 pathway hyperactivation and a potent induction of apoptosis. Recently, it has been demonstrated that PDGRFβ–AKT pathway contributes to the development of cisplatin resistance in TGCTs [138]. PTEN, which can inhibit this pathway, is lost in 50% of TGCTs [139] whereas overactivation of AKT has been observed in cisplatin-sensitive testicular tumor cells compared with their corresponding sensitive cells as a result of PDGFR-b (platelet-derived growth factor receptor beta) and PDGF-b ligand mRNA and protein levels increase [138]. No effect has been shown on activated ERK levels [138]. Interestingly, the sensitivity to cisplatin also relies on the p-AKT levels in the cells since in TGCTs a precise correlation between p-AKT levels and cell viability upon cisplatin treatment exists. In fact, phospho-AKT levels (serine 473 or threonine 308) are greater in cisplatin-resistant cells than in normal cells, whereas there are no differences in total AKT protein levels between normal and cisplatin-resistant cells [139]. PDGFRβ shRNA treatment leads CDDP-resistant cells to behave like sensitive cells. Moreover, cisplatin-resistant cells result more sensitive to PDGFRβ inhibitors (i.e. sunitinib pazopanib) action than sensitive cells, suggesting that these resistant cells strongly depend on this signaling pathway [138]. Resistant cells recover their sensitivity to CDDP when levels of p-AKT are reduced by PI3K inhibitor Ly294002, suggesting that p-AKT may be an essential player for CDDP resistance in testicular tumor cells, following the signal pathway regulated by PDGFRβ [138]. Interestingly, no correspondence between resistant or refractory TGCTs and PDGFRβ expression has been found. Only in tumors with the choriocarcinoma component, the resistance to cisplatin treatment has been correlated with higher expression of PDGFRβ [138]. Probably, other signaling pathways (i.e. PDGFRα, c-KIT) could contribute to AKT activation in other testicular tumors subtypes. As a result of PI3K activation, AKT phosphorylation takes place, ultimately leading to phosphorylation and activation of MDM2, and phosphorylation of p21, which thereby gets cytoplasmically translocated inducing cell cycle arrest and then protecting cancer cells from cisplatin-induced apoptosis.

Additional regulators of important signaling pathway have been involved in the cisplatin response. In fact, blocking of MAP–ERK kinase leads to cellular protection against cisplatin-induced apoptosis in TGCT cell lines [140]. In addition, mitogen-activated protein kinase 15 (MAPK15), the last identified member of the MAP kinase family, play an important role in promoting cell proliferation and preventing DNA damage in male germ cell tumors [141]. Overexpressed in seminomas and embryonal carcinomas and involved in important biological processes (regulation of telomerase activity, maintenance of genomic integrity, autophagy), MAPK15 has been reported to protect from ROS accumulation and DNA damage, therefore preventing p53 activation and p53-mediated cell cycle arrest, and thus favoring NTera2/D1 GCT-derived cell lines proliferation and tumorigenicity [141]. MAPK15 upregulation has been proposed as a new mechanism by which TGCT cells may restrain DNA damage response-mediated p53 activation, thus promoting their own growth and tumorigenicity. Its knockdown may negatively impact on cell growth by increasing genomic instability, thus triggering p53-dependent DNA damage response pathway and leading to cell cycle arrest and eventually to DNA repair or, alternatively, to cell death, depending on the damage intensity [141]. Thus, following cytotoxic stress, MAPK15 may function both as p53 downstream effector, inducing pathways taking part in damage protection and recovery (e.g. autophagy), and as a negative feedback mechanism on p53 itself. Indeed, MAPK15, by inducing PCNA stabilization [142] and avoiding ROS generation may facilitate to prevent DNA damage, thus providing to shut down the DNA damage response.

## CONCLUSIONS

Genetic and epigenetic events, along with environmental factors, play an important role in testicular cancer initiation and development. TGCTs often are curative disease, even in advanced stages, when treated with cisplatin-based chemotherapy, due their embryonic origin. However, significant short- and long-term toxicities can occur with consequent negative impact on the quality life of these young patients. In addition, despite the high sensitivity to cisplatin-based chemotherapy, a small fraction of patients relapses and shows resistance to this treatment. Thus, the deep knowledge of the molecular mechanisms underlying the development of TGCTs may provide new specific tools not only to develop less toxic regimens and new treatment modalities for patients with metastatic cancers, including those of somatic origin, but also to target neoplastic cells, then contributing to overcome acquired and/or intrinsic chemotherapy resistance.
